# Discontinuation from Antiretroviral Therapy: A Continuing Challenge among Adults in HIV Care in Ethiopia: A Systematic Review and Meta-Analysis

**DOI:** 10.1371/journal.pone.0169651

**Published:** 2017-01-20

**Authors:** Hailay Abrha Gesesew, Paul Ward, Kifle Woldemichael Hajito, Garumma Tolu Feyissa, Leila Mohammadi, Lillian Mwanri

**Affiliations:** 1 Public Health, Flinders University, Adelaide, Australia; 2 Epidemiology, Jimma University, Jimma, Ethiopia; 3 Joanna Briggs Institute, Adelaide University, Adelaide, Australia; 4 Department of Health Education and Behavioral Sciences, Jimma, Ethiopia; 5 Gus Fraenkel Medical Library, Flinders University, Adelaide, Australia; International AIDS Vaccine Initiative, UNITED STATES

## Abstract

**Background:**

Discontinuation of antiretroviral therapy (ART) reduces the immunological benefit of treatment and increases complications related to human immune-deficiency virus (HIV). However, the risk factors for ART discontinuation are poorly understood in developing countries particularly in Ethiopia. This review aimed to assess the best available evidence regarding risk factors for ART discontinuation in Ethiopia.

**Methods:**

Quantitative studies conducted in Ethiopia between 2002 and 2015 that evaluated factors associated with ART discontinuation were sought across six major databases. Only English language articles were included. This review considered studies that included the following outcome: ART treatment discontinuation, i.e. ‘lost to follow up’, ‘defaulting’ and ‘stopping medication’. Meta- analysis was performed with Mantel Haenszel method using Revman-5 software. Summary statistics were expressed as pooled odds ratio with 95% confidence intervals at a p-value of <0.05.

**Results:**

Nine (9) studies met the criteria of the search. Five (5) were retrospective studies, 3 were case control studies, and 1 was a prospective cohort study. The total sample size in the included studies was 62,156. Being rural dweller (OR = 2.1, 95%CI: 1.5–2.7, I^2^ = 60%), being illiterate (OR = 1.5, 95%CI: 1.1–2.1), being not married (OR = 1.4, 95%CI: 1.1–1.8), being alcohol drinker (OR = 2.9, 95%CI: 1.9–4.4, I^2^ = 39%), being tobacco smoker (OR = 2.6, 95%CI: 1.6–4.3, I^2^ = 74%), having mental illness (OR = 2.7, 95%CI: 1.6–4.6, I^2^ = 0%) and being bed ridden functional status (OR = 2.3, 95%CI: 1.5–3.4, I^2^ = 37%) were risk factors for ART discontinuation. Whereas, having HIV positive partner (OR = 0.4, 95%CI: 0.3–0.6, I^2^ = 69%) and being co-infected with Tb/HIV (OR = 0.6, 95%CI: 0.4–0.9, I^2^ = 0%) were protective factors.

**Conclusion:**

Demographic, behavioral and clinical factors influenced ART treatment discontinuation. Hence, we recommend strengthening decentralization of HIV care services in remote areas, strengthening of ART task shifting, application of seek-test-treat-succeed model, and integration of smoking cession strategies and mental health care into the routine HIV care program.

## Background

Since its emergence in the 1980s, the human immunodeficiency virus (HIV) has infected people of all ages, sexes, races and income status, leading to poor health and socio-economic outcomes across the world[1]. Since recognition of the acquired immune deficiency syndrome (AIDS) epidemic, almost 78 million people have been infected and about half of these people have died[2]. By the end of 2015, globally, 38.8 million (37.6–40.4 million) people were living with HIV[3].

Africa, Asia and Latin America were the major continents affected by the disease[4]. Sub-Saharan Africa (SSA) is the home for 76% of the global morbidity and 75% of the global mortality[3]. In 2015, Ethiopia had 39, 140 new HIV infections, 768, 040 people living with HIV, and 28, 650 HIV/AIDS deaths [3].

The advent of anti-retroviral therapy (ART), known to prolong the life of HIV patients, was a significant achievement[5]. If the quality of life and survival of people living with HIV (PLHIV) are to be improved, further effort needs to be made to ensure ART retention and its positive outcomes[6]. Discontinuation from ART (hereon in referred to as discontinuation) is the major contributor to attrition, and further to poor quality of life and death [7–13]. Discontinuation is defined as interruptions to ART due to LTFU, defaulting, transferring out and stopping medication while remaining in care[14]. Discontinuation reduces the immunological benefit of treatment and increases HIV-related complications, including AIDS-related re-admission, morbidity, mortality and drug resistance [14–19].

Discontinuation is known to be a significant problem across the globe[20–22], and Ethiopia is no exception. Studies conducted in Aksum St Marry Hospital[8], Mizan Aman General Hospital[10], Jimma University Specialized Hospital[23] and University of Gondar[24] reported that the proportion of LTFU was 9.8%, 26.7%, 28% and 31.4%, respectively. Additionally, a retrospective study from Ethiopia reported that retention of patients in care was a major challenge and varied across health facilities[25].

Primary studies conducted in Ethiopia reported socio-demographic, behavioral, clinical and institutional factors as contributors to discontinuation[7–10]. However, different studies showed conflicting association, and the existence of additional factors challenging interventions. Furthermore, the risk factors for discontinuation are still poorly understood in many developing countries including Ethiopia.

The absence of a clear and uniform definition of discontinuation is also another challenge. A study from five East African countries revealed the existence of 14 different definitions of ART defaulting were in use[26]. Currently, the definition of LTFU in Ethiopia is also not uniform, and has included a patient discontinuing from ART for more than one[8], two[9], three[10,27–29] or twelve[30] months. Additional studies have considered a ‘defaulter’ when a patient discontinues from ART for more than two months [7,23].

Until a better understanding of these risk factors is gained, attempts to increase retention rates will be ad hoc and likely to be cost ineffective. As far as is known, there is no published systematic review and meta-analysis on this topic. Additionally, the lack of high quality data on the association between discontinuation and its risk factors is a challenge preventing national HIV/AIDS control programs from providing accurate data to inform tailored intervention strategies. This study examined risk factors for discontinuation from ART among PLHIV adults in Ethiopia.

## Methods and Participants

This review has been reported using PRISMA reporting guidelines for systematic review[31] (S1 Table).

### Study protocol

A protocol for this study has been published elsewhere[32].

### Study design

A systematic review and meta-analysis was performed on studies conducted in English language in Ethiopia between 2002 and 2015. We selected 2002 as a start date for the search because this was when ART has been introduced in Ethiopia.

### Types of participants

The detail of the study participants has been described in the published protocol[32].

### Types of exposures

The review considered studies that examined risk factors for discontinuation including: age, sex, educational status, place of residence and matrimonial status, disclosure, partner’s HIV status, mental status, smoking tobacco and drinking alcohol, tuberculosis HIV (Tb/HIV) co-infection, isoniazid (INH) prophylaxis provision, cotrimoxazole or opportunist infection (OI) prophylaxis provision, presence of side effects, baseline CD4 counts, baseline WHO clinical stage, baseline functional status, baseline body mass index (BMI) level, baseline hemoglobin level and regimen substitution, distance from the facility and facility type.

### Types of outcome measures

The review considered studies that included discontinuation. Patients were considered ‘discontinued’ when they had been on ART and had missed at least one clinical appointment (one month) but had not yet been classified as “dead” or “transferred out”, or when they had stopped treatment due to any reason while they have remained in care.

### Search methods for identification of studies

An initial limited search of Google Scholar, MEDLINE, CINAHL and SCOPUS was undertaken followed by an analysis of the text words contained in the title and abstract, and of the index terms used to describe the article. A second search using all identified keywords and index terms was undertaken across the following databases: MEDLINE, PubMed, CINAHL, SCOPUS, ProQuest and Web of Science. Finally, bibliographies of all articles were reviewed to identify for additional relevant studies. Studies published in English between 2002 and 30 December 2015 were considered for inclusion in this review. The key words for this review included discontinuation, LTFU, defaulting, retention, attrition, stopping medication, interruption and Ethiopia. Full search strategy can be found in S1 Table.

### Selection of studies and quality appraisal

**Fig 1:**
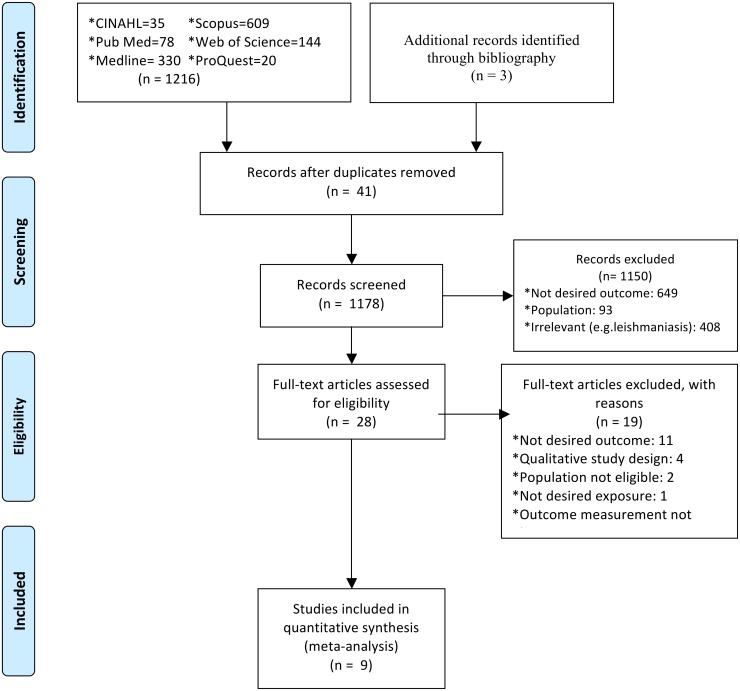
PRISMA 2009 flow diagram. This figure presents the results of the systematic search and reasons of exclusion.

The types of studies to be included in the review has been described in the published protocol[32]. The selected papers were assessed by two independent reviewers, HAG and GTF, for methodological validity prior to inclusion in the review using standardized critical appraisal instruments from the Joanna Briggs Institute Meta-Analysis of Statistics Assessment and Review Instrument (JBI-MAStARI) (S1 doc, S2 Table). Any disagreements between the reviewers were resolved through discussion. The appraisal form comprises 9 questions about the quality of the study for which articles receive values representing the extent to which they met the following criteria: Yes, No, Unclear and Not applicable. For cohort studies, appraisal based on "has bias been minimized in relation to selection of cases and of controls" was interpreted as "has bias been minimized in relation to selection of exposed and of unexposed adults living with HIV/AIDS". Risk of bias was also assessed based on Agency for Healthcare Research and Quality (AHRQ) criteria[33]. Authors of primary studies were contacted to clarify missing or unclear data. Articles were retained if at least one search term for the outcome concept was found. Articles that did not meet all eligibility criteria were excluded and reasons were noted (Fig 1).

### Data extraction

The data extraction procedure has been described in the published protocol[32]. Authors of five studies were contacted via e-mail and requested to extract row by column tables: number of patients being reported discontinuation from ART treatment vs. not, and exposures of interest.

### Data syntheses

The quantitative data were abstracted into an Excel 2007 spreadsheet and included details of study design, outcome and its measurement, sample size, number of participants with and without the event by the exposures of interest and summary of the study. Clinical heterogeneity was assessed by the authorship team and was acceptable to add each outcome to meta-analysis. Statistical heterogeneity was assessed statistically using the standard Chi-square and I^2^ tests, with significant heterogeneity detected at the P value < 0.05. Meta-analyses were conducted separately for discontinuation and each exposure of interest using RevMan-5 Software[34]. Meta-analysis was considered if I^2^ was below 85%[35]. Mantel Haenszel statistical method was used to calculate effect sizes, and forest plots to describe for the meta-analyses of exposures of interest with the event.

Pooled odds ratio (OR)[36] estimates and their 95% confidence intervals (CI) were calculated using random or fixed effect meta-analysis based on the degree of heterogeneity[35]. However, when the number of studies that reported the exposure of interest was small (n<5), only fixed effect model was considered irrespective of the level of heterogeneity[37,38]. Pooling was considered when at least two studies assessed the outcome and the exposure of interest. Publication bias was assessed using funnel plot.

## Results

### Description of articles

One thousand two hundred and nineteen (1219) potential studies including from literature search (1216) and bibliographic review (3) were identified. Fig 1 reports the results of the search and reasons of exclusion. A total of nine studies were included to assess the association between discontinuation and at least one of the aforementioned exposures of interest.

Table 1 presents the main characteristics and outcomes of reviewed studies[7–10,23,28–30,39]. Studies were conducted from across Ethiopia and the majority of them were from the northern (4) and southern (3) part of the nation. All studies had relatively high sample size and the total sample size was 62,156. The studies were analytical in type including: three case control studies[7,9,23], five retrospective cohort studies[8,10,29,30,39] and one prospective cohort study[28]. The majority of the studies (n = 7)[8–10,28–30,39] assessed factors associated with LTFU and the remaining two studies[7,23] assessed defaulting. One study that assessed LTFU[28] also assessed ‘stopped treatment’.

**Table 1:** Characteristics of included articles (n = 9).

Author	Year	Sample size (n)	Study design	Outcome of interest	Measurement	Setting	Summary
Deribe et al.[23]	2008	1094	Case control	Defaulting	Individuals who had missed two or more clinical appointments (i.e. had not been seen for the last two months)	Jimma, South west Ethiopia	Not taking hard drugs (cocaine, cannabis and IV drugs) (AOR = 0.02, 95%CI: 0.003–0.17), excessive alcohol consumption (AOR = 6, 95%CI: 3.3–11.1), being bedridden (AOR = 5.7, 95%CI: 1.6–20.2), living outside Jimma town (AOR = 2.2, 95%CI: 1.4–3.5) and having an HIV negative (AOR = 3.5, 95%CI: 1.1–11.1) or unknown (AOR = 1.7, 95%CI: 1.02 = 2.9) HIV status partner were associated with defaulting ART.
Asefa et al.[7]	2013	236	Case control	Defaulting	Cases were individuals who had missed two or more clinical appointments (i.e. had not been seen for the last two months)	Nekemtie, South west Ethiopia	Living far from the facility (AOR = 4.1, 95%CI: 1.86–9.42), being dependent for source of food (AOR = 13.9, 95%CI: 4.23–45.99], not being mentally at ease (AOR = 4.7, 95%CI: 1.65–13.35], having HIV negative partner (AOR = 5.1, 95%CI: 1.59–16.63), having a partner who hadn’t been tested for HIV or unknown (AOR = 2.8, 95%CI: 1.23–6.50] and fear of stigma (AOR = 8.3, 95%CI: 2.88–23.83) had statistically significant association with LTFU compared to their counterparts.
Wubshet et al.[39]	2013	2461	Retrospective cohort	LTFU	Adult patients who were three months late for their appointment to pick-up their antiretroviral drugs	Gondar, Northwest Ethiopia	Reasons for non-deaths losses include: stopping antiretroviral treatment due to different reasons, 135(53.36%), and relocation to another antiretroviral treatment program by self- transfer, 118(46.64%).
Berheto et al.[10]	2014	2133	Retrospective cohort	LTFU	Not taking ART refill for a period of three months or longer from the last attendance and not yet classified as ‘dead’ or ‘transferred-out’	Mizan, Southwest Ethiopia	Patients with regimen substitution (HR = 5.2, 95% CI: 3.6–7.3), non-isoniazid (INH) prophylaxis (HR = 3.7, 95% CI: 2.3–6.2), adolescent (HR = 2.1, 95% CI: 1.3–3.4), and had a baseline CD4 count < 200 cells/mm3 (HR = 1.7, 95% CIs: 1.3–2.2) were at higher risk of LTFU. WHO clinical stage 3 (HR = 0.6, 95% CIs: 0.4–0.9) and 4 (HR = 0.8, 95% CI: 0.6–1.0) patients at entry were less likely to be LTFU than clinical stage 1 patients
Tadesse et al.[8]	2014	520	Retrospective cohort	LTFU	Patients who had missed one or more clinical appointments	Axum, Northern Ethiopia	The independent predictors of LTFU of patient were being smear positive pulmonary Tb (AHR = 2.05, 95% CI: 1.02, 4.12), male gender (AHR = 2.73, 95%CI: 1.31, 5.66), regiment AZT-3TC-NVP (AHR = 3.47, 95%CI: 1.02,11.83) and weight ≥60kg (AHR = 0.24, 95% CI: 0.06,0.96).
Bucciardini et al.[28]	2015	512	Prospective cohort	LTFU^1^, Stopped treatmen^2^	^1^patients who missed scheduled visit to the same health facility more than three months after the last visit; ^2^patients known to have discontinued ART for any reasons	South Tigray, North Ethiopia	Active Tb (HR = 1.72, 95% CI: 1.23–2.41) and gender (HR = 1.64, 95% CI: 1.10–2.56) were also significantly associated with attrition.
Dessalegn et al.[9]	2015	727	Case control	LTFU	Patients who had missed two or more clinical appointments	Wukro, Northern Ethiopia	Presence of bereavement concern (AOR = 0.1, 95%CI: 0.01–0.3), not being provided with isoniazide prophylaxis (AOR = 3.04, 95%CI: 1.3–7.3), and presence of side effects (AOR = 12.3, 95%CI: 4.9–31.4) were found to be associated with increased odds for being LTFU
Melaku et al.[30]	2015	53,300^a^	Retrospective longitudinal	LTFU	If patients were not recorded as dead, transferred, or initiating ART, and if they did not have a recorded visit for 12 months or more with no subsequent visit	Ethiopia	Younger age, female gender, never being married, no formal education, low CD4+ cell count, and advanced WHO clinical stage were associated with increased LTFU
Teshome et al.[29]	2015	1173	Retrospective cohort	LTFU	If he or she failed to visit the health facility for more than 3 months after the last appointment date.	Southern, Nations, Nationalities and Peoples Region, South Ethiopia	The competing-risk regression model showed that body mass index > = 18.5 vs <18.5(AHR = 0.6, 95%CI: 0.4–0.9), WHO clinical stage late vs early (AHR = 1.4, 95%CI: 1.02–1.9), isoniazid prophylaxis no vs yes (AHR = 1.9, 95%CI = 1.1–3.2), age 26–39 vs 15–25 years (AHR = 0.6, 95%CI: 0.4–0.8), facility type health center vs hospital (AHR = 0.7, 95%CI: 0.5–0.9), and educational status 2^0+^ vs no (AHR = 0.6, 95%CI: 0.4–0.7) were independently associated with LTFU.

### Methodological quality

Three case-control studies[7,9,23] met seven out of nine JBI critical appraisal criteria, and six cohort studies[8,10,28–30,39] met eight out of nine JBI critical appraisal criteria. S2 Table presents outcome of the quality appraisal of each studies.

In addition, summary of risk of bias of the included studies was assessed based on Agency for Healthcare Research and Quality (AHRQ) criteria (S3 Table). The extent of risk bias was almost similar, and the studies had ‘low risk’ bias in the majority of areas. Due to inapplicability of design nature of the studies, they had ‘unclear risk’ judgment in a few criteria assessing the bias.

### Measurement of discontinuation from ART

Measures of discontinuation were based on LTFU, defaulting or stopping medication. Four studies[10,28,29,39] considered LTFU when HIV positive patients on ART treatment had missed three or more monthly clinical appointments and not yet been classified as “dead” or “transferring out”. One study[8] measured LTFU when adult patients were one month late for their appointment to pick-up their antiretroviral drugs whereas one other study[9] defined LTFU when patients had missed two or more clinical appointments. Another study[30] defined LTFU if they did not have a records of patients’ visit for 12 months or if there were no more subsequent visit.

The remaining two studies[7,23] measured defaulting, and both considered ‘defaulter’ for individuals who had missed two or more clinical appointments. One study[28] assessed ‘stopped treatment’ and defined ‘stopped treatment’ when HIV positive patients who have been on ART treatment but have stopped treatment due to any reason while they remained in care.

### Factors associated with discontinuation from ART among adults living HIV/AIDS

#### Socio-demographic determinants

The following socio-demographic factors were analyzed to assess their relationship with discontinuation: age, sex, place of residence, marital status and educational status. All studies assessed the relationship of age with discontinuation. All studies have measured the association of age and discontinuation, and 3 studies[10,30,39] found that patient’s age had significant association with discontinuation. Similarly, all studies have assessed the relationship between sex and discontinuation, and four studies[8,28,30,39] found a significant association. Two[23,39] of the four studies[7,9,23,39] that assessed the association between place of residence and discontinuation reported a significant association. Out of the six studies[7–9,23,29,30] that assessed correlation between marital status and discontinuation, only Melaku and colleagues [30] reported significant association. Seven studies[7–9,23,28–30] assessed the association between educational status and discontinuation, and only Melaku and colleagues [30] found statistical association.

#### Behavioral determinants

The following behavioral factors were the reported to be influential to discontinuation: disclosure, partner’s HIV status, mental status, smoking tobacco and drinking alcohol. Asefa and colleagues [7] and Deribe and colleagues [23] discussed the association of tobacco use with discontinuation, however their odds were non-significant. Both studies also assessed the correlation of alcohol with discontinuation, of which Deribe and colleagues found a statistical difference. Two [7,23] of the three studies [7,9,23] that assessed association of partner’s HIV status and discontinuation observed significant association. Dessalegn and colleagues [9] and Teshome and colleagues [29] studied the association of HIV disclosure status with discontinuation, however both found non-statistical association.

#### Clinical determinants

The following clinical factors were reported about their association with discontinuation: mental status, Tb/HIV co-infection, INH prophylaxis provision, cotrimoxazole or OI prophylaxis provision, presence of side effects, baseline CD4 counts, baseline WHO clinical stage, baseline functional status, baseline BMI level, baseline hemoglobin level and regimen substitution. Asefa and colleagues [7] and Deribe and colleagues [23] reported that having mental health problem was a risk factor for defaulting, and both reported statistically significant association. Among the three studies[7,9,29] that assessed the association between ART side effects and discontinuation, Dessalegn and colleagues[9] informed statistical significance. Seven studies[7–10,23,29,39] measured the correlation between baseline functional status and discontinuation, and only Berheto and colleagues [10] and Deribe and colleagues [23] reported the statistical significance.

Of the seven studies[7,8,10,23,28,29,39] that assessed the association between Tb status or being on Tb treatment and discontinuation, three[8,28,39] studies reported statistical difference. None of the four studies[7,8,10,23] that assessed the relationship between OI treatment or cotrimoxazole prophylaxis and discontinuation reported statistical significance. All studies assessed the correlation between baseline CD4 counts and discontinuation, and two studies [10,30] found statistical significance. WHO clinical stage as a factor for discontinuation was also assessed by six studies[9,10,28–30,39], and three of them[10,29,40] reported a statistical significance. All three studies[9,10,40] that assessed the relationship between INH prophylaxis and discontinuation reported statistical significance. Berheto and colleagues [10] and Dessalegn and colleagues [9] assessed the association between ART regimen substitution and discontinuation, but only Berheto and colleagues[10] reported significant association between these variables.

#### Institutional determinants

Distance to the health care facility[7,9] and the facility type[28,29] were the reported institutional factors influencing discontinuation. Asefa and colleagues[7] reported the presence of significant association between distance and discontinuation, and Bucciardini and colleagues [28] and Teshome and colleagues [29] reported the presence of significant association between the facility type and discontinuation.

### Meta analysis of factors affecting ART discontinuation

**Fig 2:**
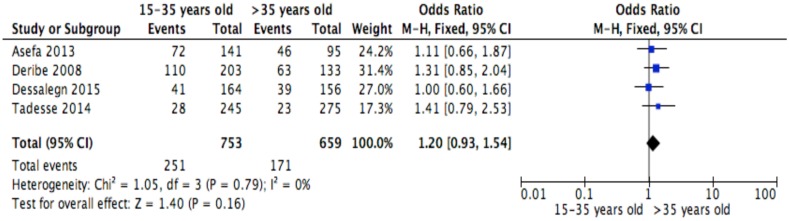
Forest plot of meta-analytic association between age and discontinuation from ART. It shows that the risk of ART discontinuation is not different by age.

**Fig 3:**
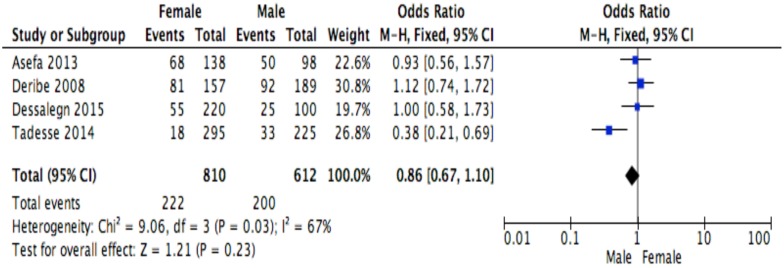
Forest plot of meta-analytic association between sex and discontinuation from ART. It shows that the risk of ART discontinuation is not different by sex.

**Fig 4:**
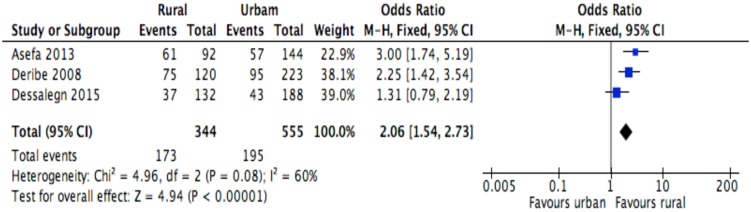
Forest plot of meta-analytic association between residence and discontinuation from ART. It shows that the risk of ART discontinuation is higher for rural than urban.

**Fig 5:**
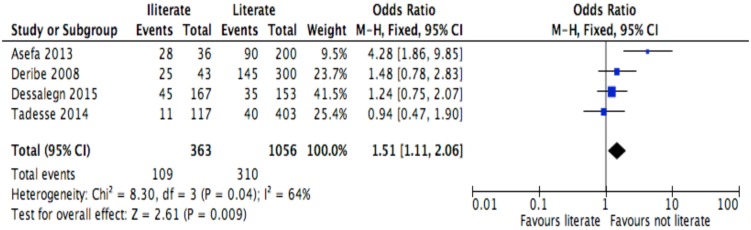
Forest plot of meta-analytic association between level of education and discontinuation from ART. It shows that the risk of ART discontinuation is higher for patients with no literacy status than literates.

**Fig 6:**
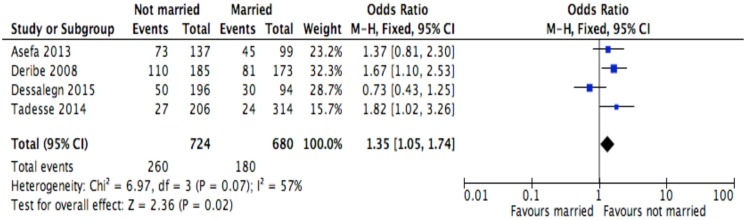
Forest plot of meta-analytic association between marital status and discontinuation from ART. It shows that the risk of ART discontinuation is higher for not-married than married.

**Fig 7:**
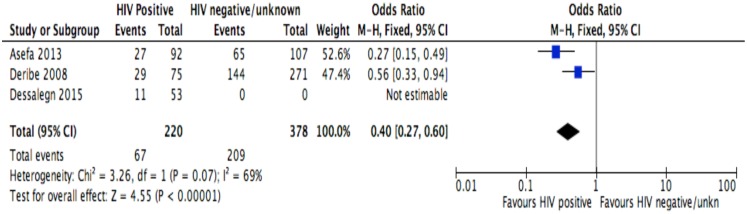
Forest plot of meta-analytic association between partners’ HIV status and discontinuation from ART. It shows that the risk of ART discontinuation is lower for patients with HIV positive partner than HIV negative/unknown partner.

**Fig 8:**
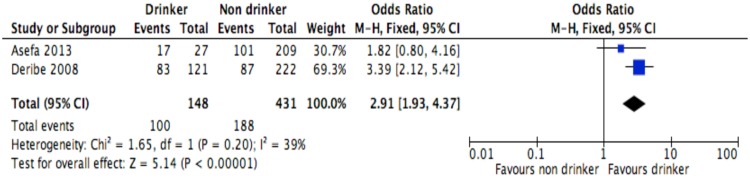
Forest plot of meta-analytic association between alcohol drinking and discontinuation from ART. It shows that the risk of ART discontinuation is higher for alcohol drinkers than non-drinkers.

**Fig 9:**
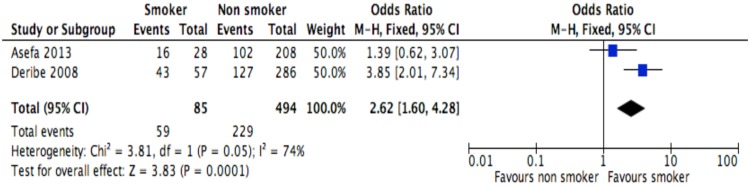
Forest plot of meta-analytic association between tobacco smoking and discontinuation from ART. It shows that the risk of ART discontinuation is higher for cigarette smokers than non-smokers.

**Fig 10:**
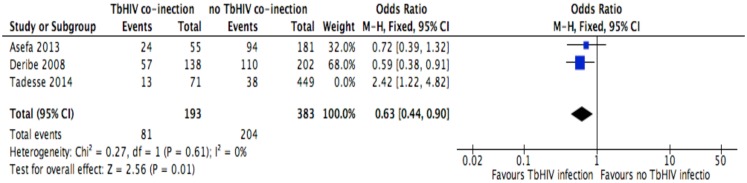
Forest plot of meta-analytic association between Tb/HIV co-infection and discontinuation from ART. It shows that the risk of ART discontinuation is lower for Tb/HIV co-infected patients than HIV alone.

**Fig 11:**
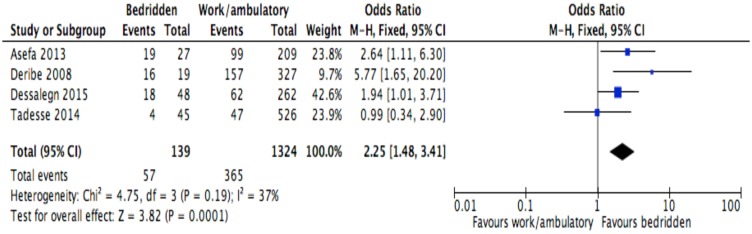
Forest plot of meta-analytic association between baseline functional status and discontinuation from ART. It shows that the risk of ART discontinuation is higher for patients with bedridden than working functional status.

**Fig 12:**
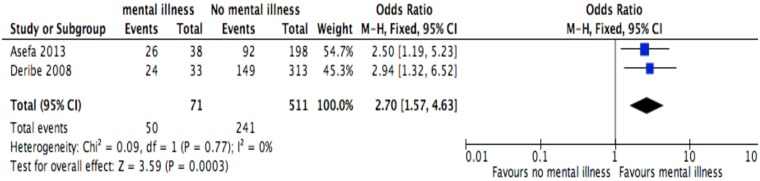
Forest plot of meta-analytic association between mental status and discontinuation from ART. It shows that the risk of ART discontinuation is higher for patients with mental status than their comparator.

**Fig 13:**
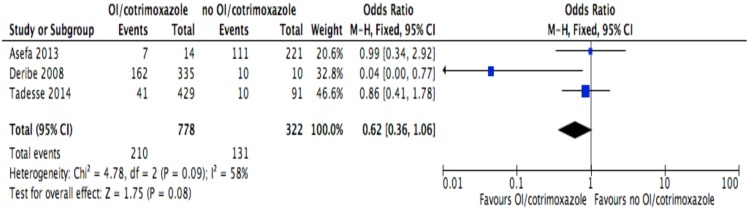
Forest plot of meta-analytic association between cotrimoxazole or opportunistic infections prophylaxis and discontinuation from ART. It shows that the risk of ART discontinuation is not different by the status of cotrimoxazole or opportunistic infections prophylaxis.

This meta-analysis identified determinants of discontinuation among adults living with HIV using proportions of the factors for the response variable assessed in primary studies[7–10,23,28–30,39]. Random effects meta-analysis model was considered for studies having moderate heterogeneity level when combined, whereas, fixed effect model was used for studies having low or no heterogeneity level[35]. However, when the number of studies reporting the exposure of interest was small (n<5), only fixed effect model was considered irrespective of the level of heterogeneity[37,38]. ART side effect was excluded from the meta-analysis because studies[7,23] reporting this variable showed severe heterogeneity (I2 = 90%). The Mantel Haenszel statistical method was used to calculate effect sizes and forest plots for the meta-analyses of socio-demographic, behavioral, clinical and institutional factors are shown in Figs 2–13.

Of the socio-demographic variables, rural dwellings (Fig 4; OR = 2.1, 95%CI: 1.5–2.7, I^2^ = 60%), no literacy status (Fig 5; OR = 1.5, 95%CI: 1.1–2.1) and being not married (Fig 6; OR = 1.4, 95%CI: 1.1–1.8) had higher odds of discontinuation than their comparator. Among the behavioral factors influencing for discontinuation, partners’ HIV positive status was found a protective factor (Fig 7; OR = 0.4, 95%CI: 0.3–0.6, I^2^ = 69%) where as alcohol drinking (Fig 8; OR = 2.9, 95%CI: 1.9–4.4, I^2^ = 39%) and tobacco smoking (Fig 9; OR = 2.6, 95%CI: 1.6–4.3, I^2^ = 74%) were found risk factors. Of the clinical factors, Tb/HIV co-infection was associated with lower odds of discontinuation (Fig 10; OR = 0.6, 95%CI: 0.4–0.9, I^2^ = 0%). Where as, having bedridden functional status (Fig 11; OR = 2.3, 95%CI: 1.5–3.4, I^2^ = 37%) and having mental illness (Fig 12; OR = 2.7, 95%CI: 1.6–4.6, I^2^ = 0%) were another risk factors. As shown in Fig 10, the article by Tadesse and colleagues [8] was removed from the meta-analysis calculation to prevent the introduction of significant heterogeneity.

## Discussion

Studies examining retention in HIV care in Ethiopia have identified discontinuation as a key challenge for patient retention[11–13]. Studies in the current systematic review and meta-analysis[7–10,23,28–30,39] have identified a number of determinants. In Ethiopia, even though a large number of HIV-infected patients discontinue after engagement with ART treatment, little research has been published as demonstrated by the low number of articles (nine studies) over a 13-year period included in this meta-analysis. This systematic review and meta-analysis identified studies conducted in three regional states of Ethiopia. The current study identified that being a rural dweller, being illiterate, being not married, being alcohol drinker, being tobacco smoker, having mental illness and being bed ridden functional status were risk factors for ART discontinuation, whereas, having HIV positive partner and being co-infected with Tb/HIV were protective factors for ART discontinuation.

The setting where the participant lived had significant influence to discontinuation with rural dwellers being more likely to discontinue compared to their urban counter parts. This finding was not a surprise as could be attributed to factors such as accessibility of the health care and availability of the transportation services[41,42]. It is therefore, plausible to hypothesise that strengthening decentralization and service integration of HIV care in remote areas would be a key for patient retention[43]. This hypothesis is also currently supported by WHO recommendations[44] of task shifting. The ART task shifting has commonly been practiced with tasks being shifted from doctors to health officers or nurses. This act has been observed to reduce patient attrition and also stated to be viable approach in rural areas. In additional to WHO recommendation, the task shifting was corroborated by a nationwide study in Ethiopia confirming that ART provision in health centers, based on health officers and nurses, is feasible, effective and acceptable[45]. Community engagement in HIV care continuum can also address the gap in inequity, particularly in rural-urban arena[46]. It is for this reason that a new model called seek-test-treat-succeed model—a model that aims at seeking out of HIV-infected individuals, offering them HIV testing and treatment, and providing support to retain—for HIV care has been promoted[47]. In addition, addressing long-term physical barriers such as roads and transportation facilities could also improve ART treatment retention[48–50].

The risk of discontinuation among patients with low literacy status was about two times higher when compared to the risk among literates. Several studies have suggested that improving knowledge of HIV care as an intervention could influence the retention of HIV positive people[51,52]. Furthermore, according to the seek-test-treat-succeed model, literate HIV infected people[53] have the capacity to provide almost 40% of HIV service-related tasks[54] and could lead to retention and re-engagement into care[47].

The risk of discontinuation among bedridden patients was two times higher when compared to the risk among working or ambulatory status. This poor baseline functional status might be due to late presentation for HIV care, a big challenge in the HIV care continuum[55]. Tobacco smokers also had high risk of discontinuation. Smoking has been noted to have a number of toxic effects that induce inflammation and weakening of the immunity, leading to failure to thrive and hindering patients from taking HIV care services continuously[56]. In addition, smokers are more likely to expose to risky sexual behaviors and this might facilitate to poor HIV/AIDS prognosis and subsequently deter from seeking HIV care services[57]. Thus, interventions for smoking cession such as Medication-Assisted Therapies (MAT) with behavioral counseling[58] and group behavior therapy programs[59] among HIV infected population should be instituted and be integrated with comprehensive HIV care.

Patients with mental illness had high probability of discontinuation than their comparator. It is well recognised that HIV and mental illness cause a serious bidirectional and synergistic combination of illness in which HIV escalates lifetime prevalence of mental illness, and mental illness increases the risk of HIV infection [60]. In addition, stigma and discriminating among HIV positive people with mental health issues can deter them from HIV care seeking[60]. This indicates the need for the inclusion of mental health into routine HIV care program.

The meta-analysis association suggests that having unknown or negative HIV partner was associated with higher odds of discontinuation than having HIV positive partner. The plausible justification might be due to negligence of counseling related to partner by health professionals[61]. It is therefore necessary to trace LTFU patients and design strict counseling for them and their partners. Additionally, it is necessary to invite patients with their partners because partners play key role in supporting patients in their HIV care continuum. Tb/HIV co-infection was associated with lower odds of discontinuation, a finding supported by a previous systematic review from sub-Saharan Africa[61]. It is plausible to hypothesize that if patients have Tb/HIV co-infection, they may attend and continue the care due to the fear of sequel of both diseases and this might have influence in retaining HIV patients in HIV care. However, further exploration is needed to examine the role of Tb/HIV co-infection in HIV care retention when compared to patients with HIV alone.

The current evidence on determinants of discontinuation has several important gaps. Measures for LTFU and defaulting were disparate to be analyzed systematically. This limitation is suggestive of weaknesses in definition of discontinuation which continues to lack a ‘gold standard’ measurement method[62]. All the studies were conducted in the three major regional states of Ethiopia named Tigray, Amhara and Oromiya in which HIV prevalence was below 2% compared to other regions such as Gambella with higher prevalence of 6.5%[63]. It is possible that regions with higher prevalence could have dissimilar risk factors for discontinuation and as such urgent attention would be warranted to establish these.

Another gap relates to the outcome status of discontinuation. Only Wubshet and colleague [39] reported the number of patients who died, survived and returned to HIV care after LTFU. Previous research reported that only 14% and 60% of LTFU patients re-engaged to HIV care at three and six months respectively[64], and those patients who re-engaged accessed the care after their health had deteriorated[65]. This shows a significant oversight for the need of future research involving the role and benefits of establishing the community-tracking system[66]. Finally, the majority of articles were retrospective cohort studies. For this reason, potential risk factors of discontinuation such as HIV related stigma were not assessed. Thus, primary studies, which may include qualitative study designs, are encouraged to explore the factors of discontinuation.

Findings of the current systematic review and meta-analysis highlight an imperative need to continue planning, implementing and evaluating intervention modalities aimed at improving retention in HIV care. To date, interventions such as reminding patients with mobile phones, text messaging and diary cards, and arranging treatment supporters have targeted the improvement of ART adherence[67,68]. Strengthening and adapting these interventions for improving patient retention could also be very effective.

Interpretations of the current study findings should consider the following important limitations. As stated, only one of the included studies in this review was prospective. This implies that meta-analytic findings can be viewed as an association and may not be causally related. The search strategy was limited to English language- a common example of reporting bias[69]. A funnel plot to detect publication bias in studies included in the meta-analysis was not reported due to the limited number of studies per each exposure (n<10)[69]. Geographic skewness and inclusion of few studies could influence the generalizability of the findings. Transferred out cases were excluded. However, we acknowledged that patients who were transferred out could continue the care in another institution resulting in overestimate of the proportion of discontinuation.

Some of the studies did not explicitly report absolute numbers of patients who discontinued by exposures of interest. Efforts to contact authors of the corresponding studies were fruitless and hence, we have been unable to report findings of meta-analytic association of the following variables: WHO clinical stage of HIV, CD4 level, regimen substitution, hemoglobin level, INH prophylaxis and facility type. We focused the systematic review on HIV positive adults, but such analysis should be followed by another work to assess risk factors for discontinuation among children. Regimen wise, studies included in the current meta-analysis were about discontinuation from first line ART treatments and this may limit the transferability of the findings to second line ART drugs.

## Conclusion

Our review identified several risk factors for ART discontinuation. Therefore, addressing the above determinants using multiple retention strategies is crucial to reduce attrition rate due to discontinuation. In addition, the retention strategies should involve multi-levels i.e. at individual-, system- and structural-level barriers.

## Supporting Information

S1 doc JBI Critical Appraisal instruments. It shows the critical appraisal checklist for each study designs.
(DOCX)

S2 doc JBI Data extraction instruments. It shows the data extraction checklist for each study designs.
(DOCX)

S1 Table Full searching strategy by databases. It shows the detailed searching strategy across data bases.
(DOCX)

S2 Table Assessment of methodological quality (n = 9). It shows the result of the methodological quality assessment.
(DOCX)

S3 Table Risk of Bias Assessment within the studies (n = 9). It shows the result of the risk bias assessment.
(DOCX)

## Abbreviations

ART: antiretroviral therapy
BMI: body mass index
HIV: human immunodeficiency virus
JBI-MAStARI: Joanna Briggs Institute Meta-Analysis of Statistics Assessment and Review Instrument
LTFU: lost to follow up
MAT: Medication-Assisted Therapies
PLHIV: people living with HIV
Tb: tuberculosis
