# Supplementary material for: Discontinuation from Antiretroviral Therapy: A Continuing Challenge among Adults in HIV Care in Ethiopia: A Systematic Review and Meta-Analysis
Source: PLoS One. 2017 Jan 20;12(1):e0169651. doi: 10.1371/journal.pone.0169651 (PMC5249214; doi:10.1371/journal.pone.0169651)
Supplement: S1 doc — It shows the critical appraisal checklist for each study designs. (DOCX) [file pone.0169651.s001.docx]

**S1 doc: JBI Critical Appraisal instruments**


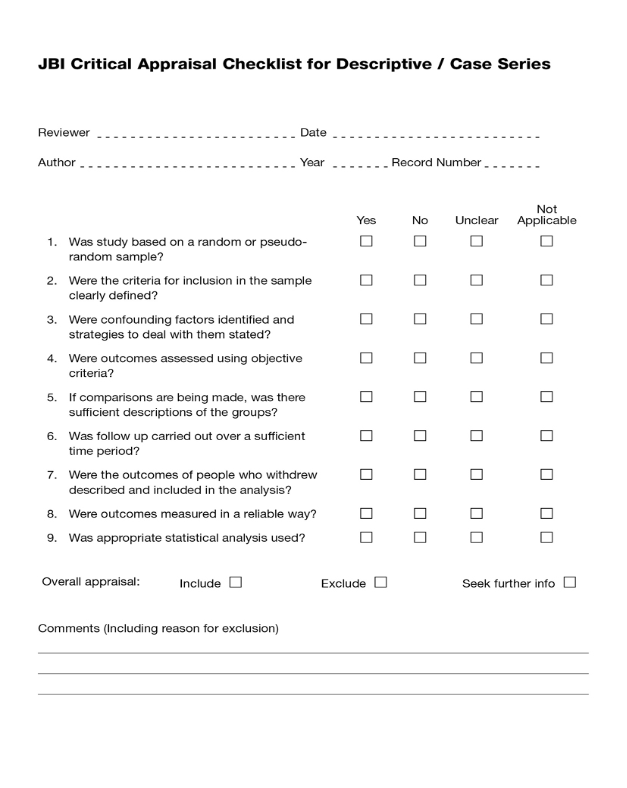


**Insert page br****this is a test message**


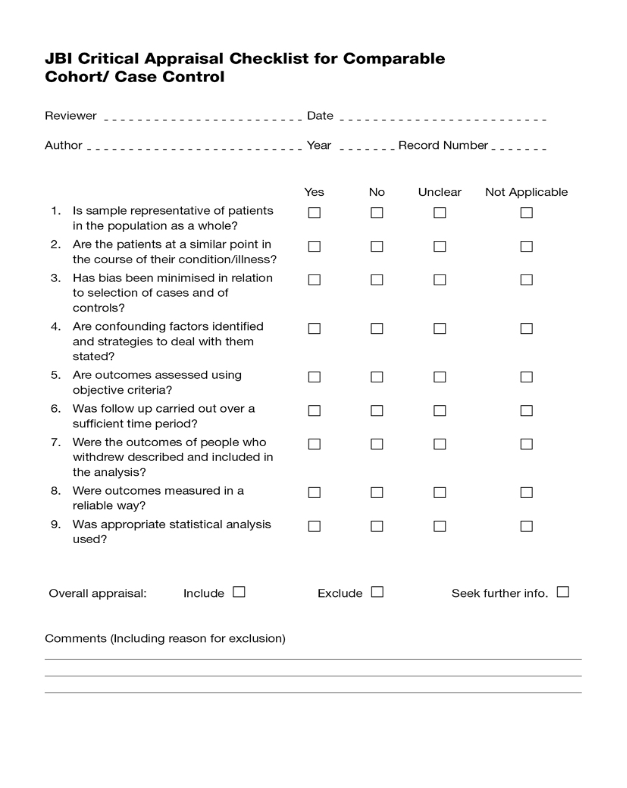


**Insert page break**
