# Supplementary figures and images for: Discontinuation from Antiretroviral Therapy: A Continuing Challenge among Adults in HIV Care in Ethiopia: A Systematic Review and Meta-Analysis
Source: PLoS One. 2017 Jan 20;12(1):e0169651. doi: 10.1371/journal.pone.0169651 (PMC5249214; doi:10.1371/journal.pone.0169651)

**S2 doc: JBI Data extraction instruments**


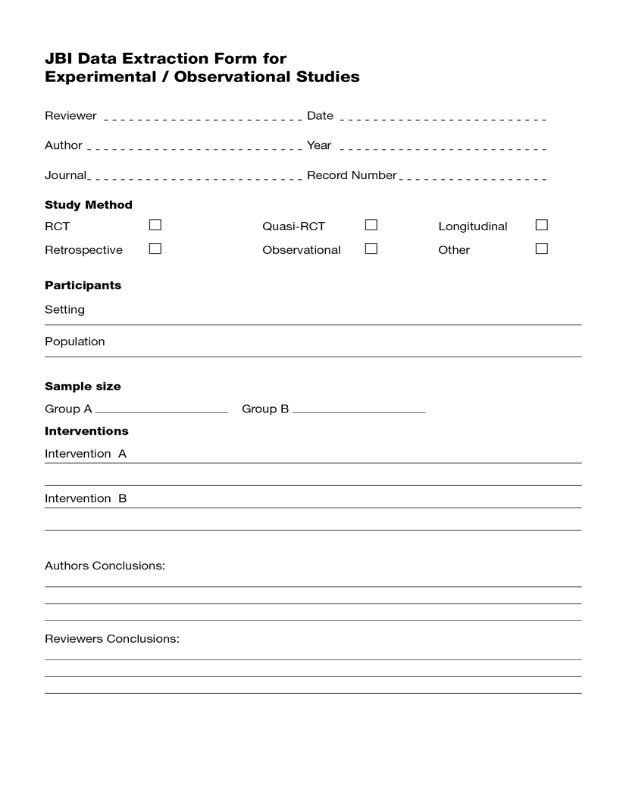


**Insert page break**


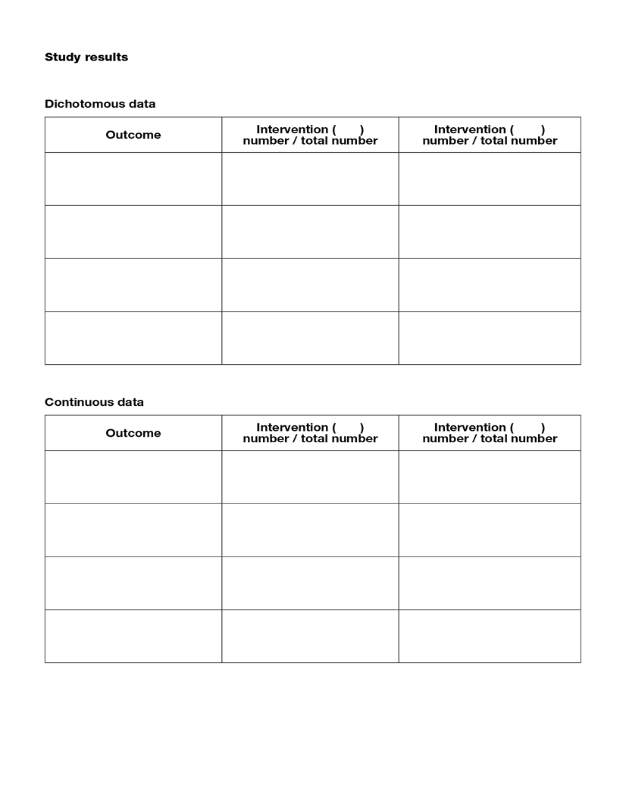

Supplement: S2 doc — It shows the data extraction checklist for each study designs. (DOCX) [file pone.0169651.s002.docx]
