# Supplementary material for: Discontinuation from Antiretroviral Therapy: A Continuing Challenge among Adults in HIV Care in Ethiopia: A Systematic Review and Meta-Analysis
Source: PLoS One. 2017 Jan 20;12(1):e0169651. doi: 10.1371/journal.pone.0169651 (PMC5249214; doi:10.1371/journal.pone.0169651)
Supplement: S1 Table — It shows the detailed searching strategy across data bases. (DOCX) [file pone.0169651.s003.docx]

**S1 Table:** **Full searching strategy by databases**

Medline searching strategy

| 1 | exp HIV Infections |
| --- | --- |
| 2 | Antiretroviral Therapy, Highly Active |
| 3 | (HIV or AIDS or HIV-AIDS or Acquired Immunodeficiency Syndrome or Human immunodeficiency virus).tw. |
| 4 | (HIV adj1 (treat* or therap* or care or medication*)).tw. |
| 5 | (antiretroviral* or anti-retroviral* or HAART or ART or anti-hiv).tw. |
| 6 | Or/1-5 |
| 7 | lost to follow up |
| 8 | (discontinu* or stop* medication* or "drop out" or default* or "los* to follow up" or LTFU or interrupt* or attrition or retention or link* or persist*).tw. |
| 9 | Or/7-8 |
| 10 | africa, eastern/ or ethiopia |
| 11 | (east* Africa* or "Horn of Africa*" or ethiopia* or Addis Ababa* or Afar* or Semera* or Amhara* or Bahir Dar* or Benishangul-Gumuz* or Asosa* or Dire Dawa* or Gambela* or Harar* or Oromia* or Somali* or Jijiga* or Hawassa* or Tigray* or Meke'ele* or "Southern Nations Nationalities and peoples region" or SNNPR).tw. |
| 12 | Or/10-11 |
| 13 | 6 and 9 and 12 |

Pub med searching strategy

| 1 | (((( HIV OR aids OR hiv-aids OR acquired immunodeficiency syndrome OR human immunodeficiency virus OR antiretroviral OR anti-retroviral OR haart ART OR anti-hiv OR (HIV AND(treatment OR therapy OR care OR medication)))) |
| --- | --- |
| 2 | (discontinue* OR stop* medication OR “drop out” OR default* OR “lost to follow up” OR ltfu OR interruption OR attrition OR retention OR link* OR persist) |
| 3 | ((east africa OR “Horn of Africa” OR ethiopia OR addis ababa OR afar OR semera OR amhara OR bahir dar OR benshangul gumz OR asosa OR dire dawa OR gambella OR harar OR oromiya OR Somali OR jijiga OR hawassa OR tigray OR meke’le OR “southern nations nationalities and peoples region) NOT Medline[sb])” LIMITED to English |
| 4 | 1 AND 2 AND 3 |

Web of Science searching strategy

| 1 | ((HIV or AIDS or HIV-AIDS or Acquired Immunodeficiency Syndrome or Human immunodeficiency virus) AND ((antiretroviral* or anti-retroviral* or HAART or ART or anti-hiv) OR (HIV NEAR/1 (treat* or therap* or care or medication*))) |
| --- | --- |
| 2 | (discontinu* or stop* medication* or "drop out" or default* or "los* to follow up" or LTFU or interrupt* or attrition or retention or link* or persist*) |
| 3 | (east* Africa* or "Horn of Africa*" or ethiopia* or Addis Ababa* or Afar* or Semera* or Amhara* or Bahir Dar* or Benishangul-Gumuz* or Asosa* or Dire Dawa* or Gambela* or Harar* or Oromia* or Somali* or Jijiga* or Hawassa* or Tigray* or Meke'ele* or "Southern Nations Nationalities and peoples region" or snnpr)) |
| 4 | 1 AND 2 AND 3;  Limited by language (English) and countries/territories (to Ethiopia) |

Proquest searching strategy

| 1 | ((HIV OR AIDS OR HIV-AIDS OR Acquired Immunodeficiency Syndrome OR Human immunodeficiency virus) |
| --- | --- |
| 2 | ((antiretroviral OR anti-retroviral OR heart OR ART OR anti-hiv) OR (HIV NEAR/1 (treat OR therapy OR care OR medication))) AND (discontinue OR stop medication OR "drop out" OR default OR "los to follow up" OR LTFU OR interrupt OR attrition OR retention OR persist) |
| 3 | (east Africa OR "Horn of Africa" OR ethiopia OR Addis Ababa OR Afar OR Semera OR Amhara OR bahr Dar OR Benishangul-Gumuz OR sosa OR Dire daiwa OR Gambela OR harare OR Oromia OR Somali OR Jijiga OR Hawassa OR Tigray OR Meke'ele OR "Southern Nations Nationalities and peoples region" OR SNNPR)) |
| 4 | 1 AND 2 AND 3; *Limited to full text, English language, b/n 2002 and 2015, location=Ethiopia, scholarly articles and conference proceedings* |

Scopus searching strategy

| 1 | ALL ((antiretroviral* OR anti-retroviral* OR haart OR art OR anti-hiv) OR (hiv W/1 (treat* OR therap* OR care OR medication*)) OR hiv OR aids OR “acquired immunodeficiency syndrome” OR “human immunodeficiency virus”) |
| --- | --- |
| 2 | ALL (discontinue* OR “stop* medication” OR “drop out” OR default* OR “lost* to follow up” OR ltfu OR interrupt* OR attrition OR retention OR link* OR persist*) |
| 3 | ALL (ethiopia OR addis ababa OR oromiya OR afar OR tigray OR amhara OR afar OR harar OR benshangul gumz OR Somali OR OR gambella OR dire dawa OR “southern nations nationalities and peoples region OR snnpr) |
| 4 | 1 AND 2 AND 3; Limited to country, Ethiopia AND Subject area medicine/sociology/psychology AND English |

CINAHL Searching strategy

| S13 | S10 AND S11 AND S12 |
| --- | --- |
| S12 | S1 OR S2 OR S3 OR S7 |
| S11 | S6 OR S9 |
| S10 | S4 OR S5 OR S8 |
| S9 | Tx discontinu* or stop* medication* or "drop out" or default* or "los* to follow up" or LTFU or interrupt* or attrition or retention or link* or persist* |
| S8 | east* Africa* or "Horn of Africa*" or ethiopia* or Addis Ababa* or Afar* or Semera* or Amhara* or Bahir Dar* or Benishangul-Gumuz* or Asosa* or Dire Dawa* or Gambela* or Harar* or Oromia* or Somali* or Jijiga* or Hawassa* or Tigray* or Meke'ele* or "Southern Nations Nationalities and peoples region" or SNNPR |
| S7 | hiv or "acquired immunodeficiency syndrome" or "HIV infections" or AIDS or "Human Immunodeficiency virus" or "antiretroviral treat*" or "highly active antiretroviral therapy or HAART or ART or HIV treat* or "anti-retroviral agent" |
| S6 | (MH "After Care") |
| S5 | (MH "Africa, Eastern") |
| S4 | (MH "Ethiopia") |
| S3 | (MH "Antiretroviral Therapy, Highly Active") |
| S2 | (MH "Acquired Immunodeficiency Syndrome") |
| S1 | (MH "HIV Infections") |
