# Supplementary material for: Discontinuation from Antiretroviral Therapy: A Continuing Challenge among Adults in HIV Care in Ethiopia: A Systematic Review and Meta-Analysis
Source: PLoS One. 2017 Jan 20;12(1):e0169651. doi: 10.1371/journal.pone.0169651 (PMC5249214; doi:10.1371/journal.pone.0169651)
Supplement: S2 Table — It shows the result of the methodological quality assessment. (DOCX) [file pone.0169651.s004.docx]

**S2 Table: Assessment of methodological quality (n=9)**

| Authors | Q1 | Q2 | Q3 | Q4 | Q5 | Q6 | Q7 | Q8 | Q9 | % |
| --- | --- | --- | --- | --- | --- | --- | --- | --- | --- | --- |
| Asefa et al. | Y | Y | Y | Y | Y | NA | N | Y | Y | 88 |
| Berheto et al. | Y | Y | Y | Y | Y | Y | NA | Y | Y | 100 |
| Bucciardini et al. | Y | Y | Y | Y | Y | Y | N | Y | Y | 89 |
| Deribe et al. | Y | Y | Y | Y | Y | NA | N | Y | Y | 88 |
| Melaku et al. | Y | Y | Y | Y | Y | Y | NA | Y | Y | 100 |
| Dessalegn et al. | Y | Y | Y | Y | Y | NA | NA | Y | Y | 100 |
| Tadesse et al. | Y | Y | Y | Y | Y | Y | NA | Y | Y | 100 |
| Teshome et al. | Y | Y | Y | Y | Y | Y | NA | Y | Y | 100 |
| Wubshet et al | Y | Y | Y | Y | Y | Y | N | Y | Y | 88 |

Q= Question Y=Yes; N= No; NA= Not applicable
