# Supplementary material for: Discontinuation from Antiretroviral Therapy: A Continuing Challenge among Adults in HIV Care in Ethiopia: A Systematic Review and Meta-Analysis
Source: PLoS One. 2017 Jan 20;12(1):e0169651. doi: 10.1371/journal.pone.0169651 (PMC5249214; doi:10.1371/journal.pone.0169651)
Supplement: S3 Table — It shows the result of the risk bias assessment. (DOCX) [file pone.0169651.s005.docx]

**S3 Table: Risk of Bias Assessment within the studies (n=9)**

| Study | Random Sequence Generation (Selection bias) | Allocation Concealment (Selection bias) | Blinding of Participants and personnel (Performance bias) | Blinding of outcome Assessment (Detection bias) | Incomplete Outcome Data (attrition bias) | Selective reporting (Reporting bias) | Other |
| --- | --- | --- | --- | --- | --- | --- | --- |
| Asefa et al. | Unclear risk^a^ | Unclear riska | Unclear risk | Low risk | Low risk | Low risk | Low risk |
| Berheto et al. | Unclear risk^a^ | Unclear risk^a^ | Unclear risk | Low risk | Low risk | Low risk | Low risk |
| Bucciardini et al. | Unclear risk^a^ | Unclear risk^a^ | Unclear risk | Low risk | Low risk | Low risk | Low risk |
| Deribe et al. | Unclear risk^a^ | Unclear risk^a^ | Unclear risk | Low risk | Low risk | Low risk | Low risk |
| Melaku et al. | Unclear risk^a^ | Unclear risk^a^ | Unclear risk | Low risk | Low risk | Low risk | Low risk |
| Dessalegn et al. | Unclear risk^a^ | Unclear risk^a^ | Unclear risk | Low risk | Low risk | Low risk | Low risk |
| Tadesse et al. | Unclear risk^a^ | Unclear risk^a^ | Unclear risk | Low risk | Low risk | Low risk | Low risk |
| Teshome et al. | Unclear risk^a^ | Unclear risk^a^ | Unclear risk | Low risk | Low risk | Low risk | Low risk |
| Wubshet et al. | Unclear risk^a^ | Unclear risk^a^ | Unclear risk | Low risk | Low risk | Low risk | Low risk |

^a^ = Not applicable due to type of study design
